# Physically based vs. data-driven models for streamflow and reservoir volume prediction at a data-scarce semi-arid basin

**DOI:** 10.1007/s11356-024-33732-w

**Published:** 2024-05-29

**Authors:** Gülhan Özdoğan-Sarıkoç, Filiz Dadaser-Celik

**Affiliations:** 1 Department of Vegetable and Animal Production, Suluova Vocational School, Amasya University, Amasya, Turkey; 2 Department of Environmental Engineering, Erciyes University, Kayseri, Turkey

**Keywords:** Hydrologic modelling, SWAT, NARX, Streamflow, Reservoir volume

## Abstract

Physically based or data-driven models can be used for understanding basinwide hydrological processes and creating predictions for future conditions. Physically based models use physical laws and principles to represent hydrological processes. In contrast, data-driven models focus on input–output relationships. Although both approaches have found applications in hydrology, studies that compare these approaches are still limited for data-scarce, semi-arid basins with altered hydrological regimes. This study aims to compare the performances of a physically based model (Soil and Water Assessment Tool (SWAT)) and a data-driven model (Nonlinear AutoRegressive eXogenous model (NARX)) for reservoir volume and streamflow prediction in a data-scarce semi-arid region. The study was conducted in the Tersakan Basin, a semi-arid agricultural basin in Türkiye, where the basin hydrology was significantly altered due to reservoirs (Ladik and Yedikir Reservoir) constructed for irrigation purposes. The models were calibrated and validated for streamflow and reservoir volumes. The results show that (1) NARX performed better in the prediction of water volumes of Ladik and Yedikir Reservoirs and streamflow at the basin outlet than SWAT (2). The SWAT and NARX models both provided the best performance when predicting water volumes at the Ladik reservoir. Both models provided the second best performance during the prediction of water volumes at the Yedikir reservoir. The model performances were the lowest for prediction of streamflow at the basin outlet (3). Comparison of physically based and data-driven models is challenging due to their different characteristics and input data requirements. In this study, the data-driven model provided higher performance than the physically based model. However, input data used for establishing the physically based model had several uncertainties, which may be responsible for the lower performance. Data-driven models can provide alternatives to physically-based models under data-scarce conditions.

## Introduction

Developing hydrological models at the basin scale is challenging due to the complexity of hydrological processes, spatial variability of soil, geology, and land use/cover characteristics, and spatial and temporal variability of climatic conditions. Hydrological models enable us to understand how physical or climatic changes could affect basinwide hydrological processes and predict basin response to natural or artificial changes (Gupta et al. 2005; Rajat 2021).

There are two classes of hydrological models: physically based or data-driven models. Physically based models consist of mathematical equations that are representation of conceptual models and physical laws such as conservation of mass and conservation of momentum (Chua 2012). These models have been used to predict various hydrological variables including streamflow (Ouyang et al. 2021) and reservoir volumes (Beharry et al. 2021), and explain rainfall-runoff relationships (Liu and Todini 2002) and assess hydrological impacts of global circulation patterns (Liang et al. 1994), and determine occurrence of floods (Borah 2011; Costabile et al. 2013; Costabile and Macchione 2015). The processing of hydrological parameters for the development of physically based models requires expertise, high-quality data, and detailed knowledge of the basin processes (Kim et al. 2015a). Additionally, some studies noted that site-specific constraints and the challenges in reaching input data contributed to some of the short-term prediction errors of physically based models (Costabile and Macchione 2015). Physically based models are capable of explaining the physical processes underlying hydrological events.

Data-driven models have also been used for prediction and forecasting in hydrological studies (Barzegar et al. 2021; Elshorbagy et al. 2010; Evora and Coulibaly 2009; Hu et al. 2021; Ouyang et al. 2021; Özdoğan-Sarıkoç et al. 2023; Tsai et al. 2015; Yaseen et al. 2015; Zhang et al. 2018). These models became more popular with the advances in computation techniques and capacities over the last decades. Data-driven models can be trained easily without knowledge about the physical processes in the basin, offering a valuable tool for modelling difficult or complex terrains with data limitations (Wunsch et al. 2018). These models can be more quickly developed with minimum inputs (Mosavi et al. 2018). However, they have been faced criticism for their inherent lack of transparency and difficulty in reproducing results (Elshorbagy et al. 2010). For example, some of the data-driven models, such as artificial neural networks (ANNs), adapt black-box approach, where inputs are related to outputs using various transfer functions without using knowledge about physical relationships (Kanungo et al. 2006).

Todini (2007) emphasized that an objective comparison is necessary to evaluate the uncertainties and advantages of physically based and data-based models. However, studies that compared these two approaches are still very limited. We provide a summary of previous studies, listed in the Web of Science index, that compared physically based and data-driven models in hydrological applications in Table 1. The studies listed in Table 1 were conducted in different locations, with different models, and with data sets having different characteristics. The annual precipitation in these watersheds ranged from 660 to 2715 mm and studies in arid and semi-arid landscapes were limited. Performance evaluation was done based on various hydrological variables including streamflow, flood events, and evapotranspiration. Still, model prediction performance for some hydrological variables such as reservoir volumes has not yet been investigated. Most of the studies (Ahmadi et al. 2019; Demirel et al. 2009; Kim et al. 2015b; Pradhan et al. 2020; Rabezanahary Tanteliniaina et al. 2021; Srivastava et al. 2006; Valeh et al. 2021; Zakizadeh et al. 2020) focused on the comparison of SWAT with classical ANN models (such as feed-forward networks) for streamflow forecasting. The performance of NARX, which is an ANN model, widely used in data-driven modeling of complex systems, has not been compared with SWAT for streamflow and reservoir volume forecasting. The studies that compared two approaches generally provided better performance with data-driven models (Ahmadi et al. 2019; Demirel et al. 2009; Fan et al. 2020; Hussain et al. 2021; Ji et al. 2021; Kim and Kim 2021; Kim et al. 2015b; Lee et al. 2020; Pradhan et al. 2020; Rabezanahary Tanteliniaina et al. 2021; Srivastava et al. 2006; Sungmin et al. 2020; Valeh et al. 2021; Zakizadeh et al. 2020). However, the models were compared in watersheds where hydrological system was mostly in its natural state and where high-quality data were rather accessible. The model performances have not been compared in watersheds where the hydrological processes were highly modified with reservoirs and irrigation activities and in basin where data availability poses major challenges. Additionally, to the best of our knowledge, there has been no performance comparison conducted for reservoir volume prediction. In this study, we aim to contribute to the available literature by using a physical-based model, SWAT (Arnold et al. 1998), and a data-driven model, NARX, for reservoir volume and streamflow prediction, in a semi-arid, data-scarce basin, where basin hydrology was altered through human interventions.

**Table 1:** Previous studies that compared physically-based and data-driven models for the prediction of different hydrological variables*

Basin name	Area (km^2^)	Precipitation (mm/year)	Physically based model used	Data-driven model used	Hydrological variables	Performed the best	References
Southeastern Pennsylvania, USA	47.62	1204	SWAT	ANN	Streamflow	ANN	Srivastava et al. (2006)
Pracana basin, Portugal	1433	900 to 1400	SWAT	ANN	Flow forecast	ANN	Demirel et al. (2009)
Rhine and Neckar Rivers, Germany	68,827 and 13,783	–	HM, MCM	ANN, ANFIS	River flood	ANN	Shrestha and Nestmann (2009)
Lower Mekong Basin, East and Southeast Asia	600,000	–	URBS	ANN, ANFIS	Streamflow	ANN, ANFIS	Chua (2012)
Taehwa River watershed, Korea	643.96	1274.6	SWAT	ANN, SOM	Streamflow	SOM	Kim et al. (2015a)
Limkheda watershed, India	220.86	660	SWAT	NNs	Streamflow	NNs	Makwana and Tiwari (2017)
Small Aral Sea basin, seven different countries	24,000	–	HBV, GR4J, SIMHYD	MLR, ETR, LGB, XGB	Runoff	MLR	Ayzel and Izhitskiy (2018)
Mekong river, East and Southeast Asia	–	–	SWAT	LSTM	Streamflow	LSTM	Giha et al. (2018)
Miño-Sil and Segura River Basins, Spain	843	660 to 1632	SWAT	ANN	Runoff	ANN	Jimeno-Sáez et al. (2018)
Kan watershed, Tehrab	220	–	SWAT, IHACRES	ANN	Rainfall-Runoff	ANN	Ahmadi et al. (2019)
161 catchments in 11 countries	4 to 3781	–	ECMWF, SWBM	LSTM	Runoff	LSTM	Sungmin et al. (2020)
Mahantango Creek watershed, USA	7.3	1080	SWAT-VSA	ANN, ARMA	Streamflow	SWAT-VSA, ANN	Wagena et al. (2020)
West-Seti, Rod, and Sre Pok River Basins	7438 to 39,060	100–700	SWAT	ANN	Streamflow	ANN	Pradhan et al. (2020)
Mekong River Basin, six different countries	795,000	–	SWAT	LSTM	Future Runoff	LSTM	Lee et al. (2020)
Poyang Lake Basin, China	162,225	1680	SWAT	LSTM, ANN	Runoff	LSTM	Fan et al. (2020)
Darake watershed, Iran	25	–	SWAT	ANN	Runoff	ANN	Zakizadeh et al. (2020)
49 flux tower sites in the world	–	–	SEBS	DNN, RF, SR	Evapotranspiration	RF, DNN	Hu et al. (2021)
Mangoky River, Madagascar	55,750	600	SWAT	ANN	Future Runoff	ANN, SWAT	Rabezanahary Tanteliniaina et al. (2021)
Yeongsan River basin, South Korea	3371.4	1293	SWAT	LSTM	Rainfall-runoff	LSTM	Kim and Kim (2021)
Tianshan Mountains, Central Asia	63,000	–	SWAT, SWAT_Glacier	LSTM, XGBoost, SVR	Discharge forecasting	LSTM	Ji et al. (2021)
Ammameh basin, Iran	35.06	735	SWAT	ANN	Rainfall-runoff	ANN	Valeh et al. (2021)
Fengshan Creek basin, Taiwan	250.1	1754–2715	WASH123D, HEC-HMS	SVM	Flood forecasting	SVM	Hussain et al. (2021)
*Hablehroud River, Iran*	*–*	*–*	*SWAT, IHACRES*	*ANFIS, ANN, SVM*	*Streamflow forecasting*	*ANFIS*	Ashrafzadeh et al. (2024)
*Hemavati River, India*	*5410*	*1530*	*SWAT*	*M5P*	*Rainfall-runoff*	*M5P*	Kumar et al. (2024)
*Dagu River Basin, China*	*4631*	*1466*	*SWAT*	*LSTM, GRU, EMD-LSTM, EMD-GRU*	*Runoff prediction*	*EMD-LSTM*	Yang et al. (2024)

^*^ANFIS, adaptive neuro-fuzzy inference system; *ANN*, artificial neural network; *ARMA*, auto-regressive moving-average; *DNN*, deep neuron network; *ECMWF*, European Centre for Medium-Range Weather Forecasts; *ETR*, extra trees regression; *GR4J*, Genie Rural a 4 parametres Journalier; *HBV*, Hydrologiska Byråns Vattenbalansavdelning; *HEC-HMS*, hydrologic modeling system; *HM*, hydrodynamic model; *IHACRES*, Identification of unit hydrographs and component flows from rainfall; *LGB*, light gradient boosting machine; *LSTM*, long short-term memory; *MCM*, Muskingum Cunge Model; *MLR*, multiple linear regression; *NARX*, Nonlinear AutoRegressive eXogenous model; *NNs*, neural networks; *RF*, random forest; *RNN*, recurrent neural network; *SEBS*, the surface energy balance system; *SR*, symbolic regression; *SOM*, self-organizing map; *SVM*, support vector machine; *SVR*, support vector regression; *SWAT*, The Soil and Water Assessment Tool; *SWAT-VSA*, Soil and Water Assessment Tool-Variable Source Area model; *SWAT_Glacier*, an extended SWAT model with glacier melting mechanism; *SWBM*, simple water balance model; *URBS*, the unified run-off basin simulation; *XGB*, eXtreme Gradient Boosting machine; *XGBoost*, extreme gradient boosting; *IHACRES*, Identifcation of Hydrographs And Component fows from Rainfall, Evaporation, and Streamfow data; *GRU*, gated cycle unit; *EMD*, empirical mode decomposition

SWAT, a lump-parameter, continuous time-scale model, is among the most-used physically based models to characterize basin-scale hydrological processes and to simulate streamflow, reservoir volumes, and reservoir operations (Kim and Parajuli 2012). SWAT has been used for investigating climate change impacts (Jha et al. 2006; Narsimlu et al. 2013; Sood et al. 2013), water quality characterization (Pisinaras et al. 2010; Pohlert et al. 2005), land use/cover change impact assessment (Du et al. 2013; Marhaento et al. 2017), and testing the effects of scenarios (Pisinaras et al. 2010) and best management practices (Kaini et al. 2012; Uniyal et al. 2020). A few studies are available in the literature that used SWAT for prediction of reservoir volumes or reservoir water levels (Beharry et al. 2021, Kim and Parajuli 2012, Jouma and Dadaser‐Celik 2022, Kim et al. 2021, Sedighkia and Abdoli 2022, Zhang et al. 2011, Zhang et al. 2022).

NARX is a relatively new and special type of recurrent neural network (RNN), characterized by its utilization of feedback connections, which typically provides higher performance than conventional RNNs (Lin et al. 1996). NARX has been successfully used for modeling nonlinear systems (Wunsch et al. 2018) with its capability to store information in memory much longer than other RNNs (Lin et al. 1996), which leads to faster convergence and better generalization (Lin et al. 1998). Most researchers used NARX for predicting groundwater levels (Guzman et al. 2017, 2019; Javadinejad et al. 2020; Nunno and Granata 2020; Wunsch et al. 2018), reservoir inflows (Ghazali et al. 2018; Yang et al. 2019), streamflow (Nunno et al. 2021), water temperatures (Kwak et al. 2017), and floods (Chang et al. 2014, 2022; Nanda et al. 2016; Rjeily et al. 2017). The number of studies that focused on reservoir volume and streamflow prediction is comparatively lower (Ghazali et al. 2018; Nunno et al. 2021; Yang et al. 2019). To the best of our knowledge, no studies used NARX for reservoir volume prediction.

This study was conducted at the Tersakan Basin in Türkiye. The climatic characteristics of the Tersakan Basin is semi-arid, with about 450-mm annual precipitation. Tersakan Basin has an altered hydrological regime due to construction of reservoirs on the stream network. Another difficulty for hydrological modeling is caused by the uncertainty in the amount of water used from reservoirs for irrigation purposes. The limited availability of input data such as land use/cover and soil data also caused challenges. This study aims to compare the performances of a physically based model (SWAT) and a data-driven model (NARX) in the Tersakan Basin. The weaknesses and strengths of these approaches were discussed. The analyses in this study could help evaluate the potential of different modelling approaches in predicting reservoir volumes and streamflow in challenging watersheds. This study can also create information related to the use of a new type of ANN model, NARX, in hydrological modelling studies, which is quite limited in the literature.

## Materials and methods

**Fig. 1:**
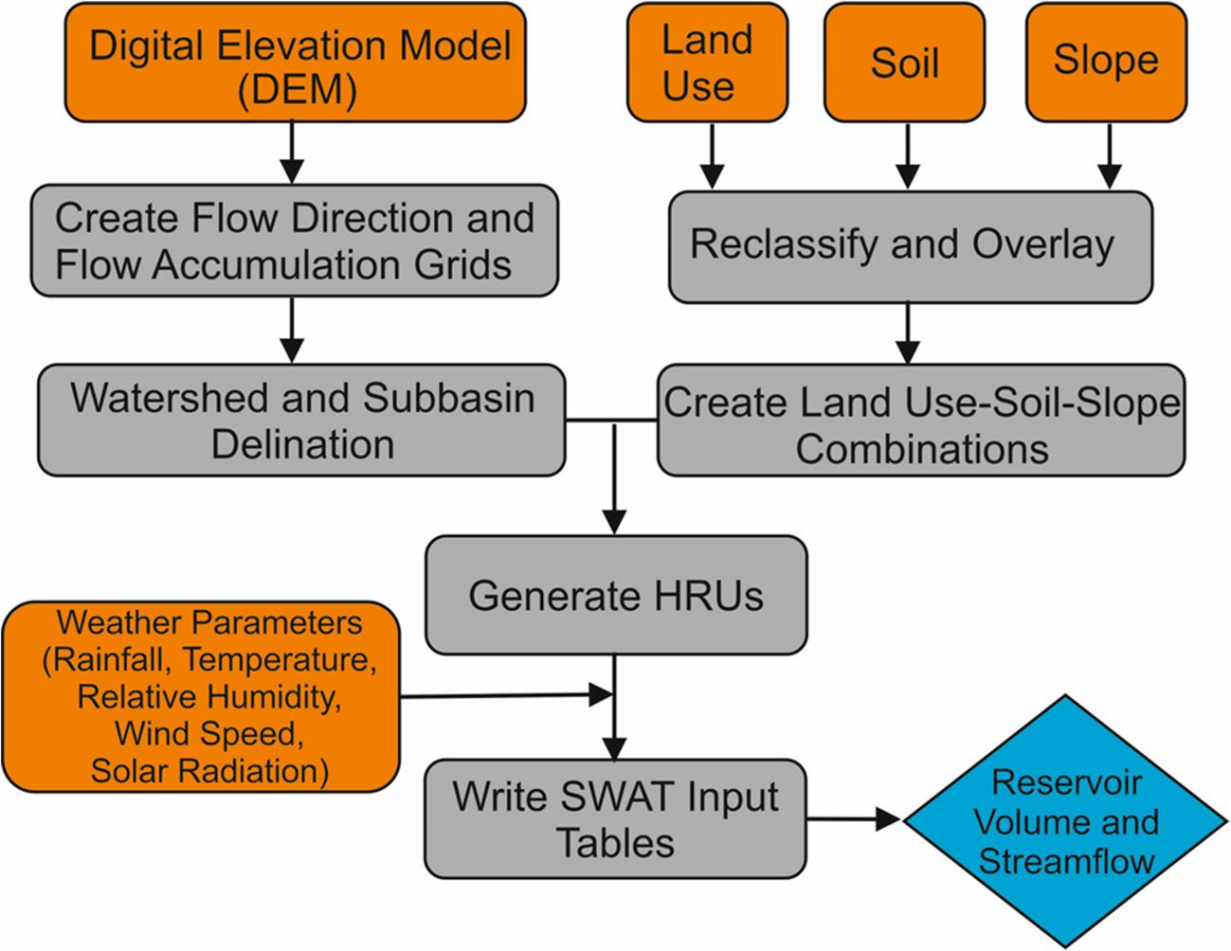
Stages of SWAT application

**Fig. 2:**
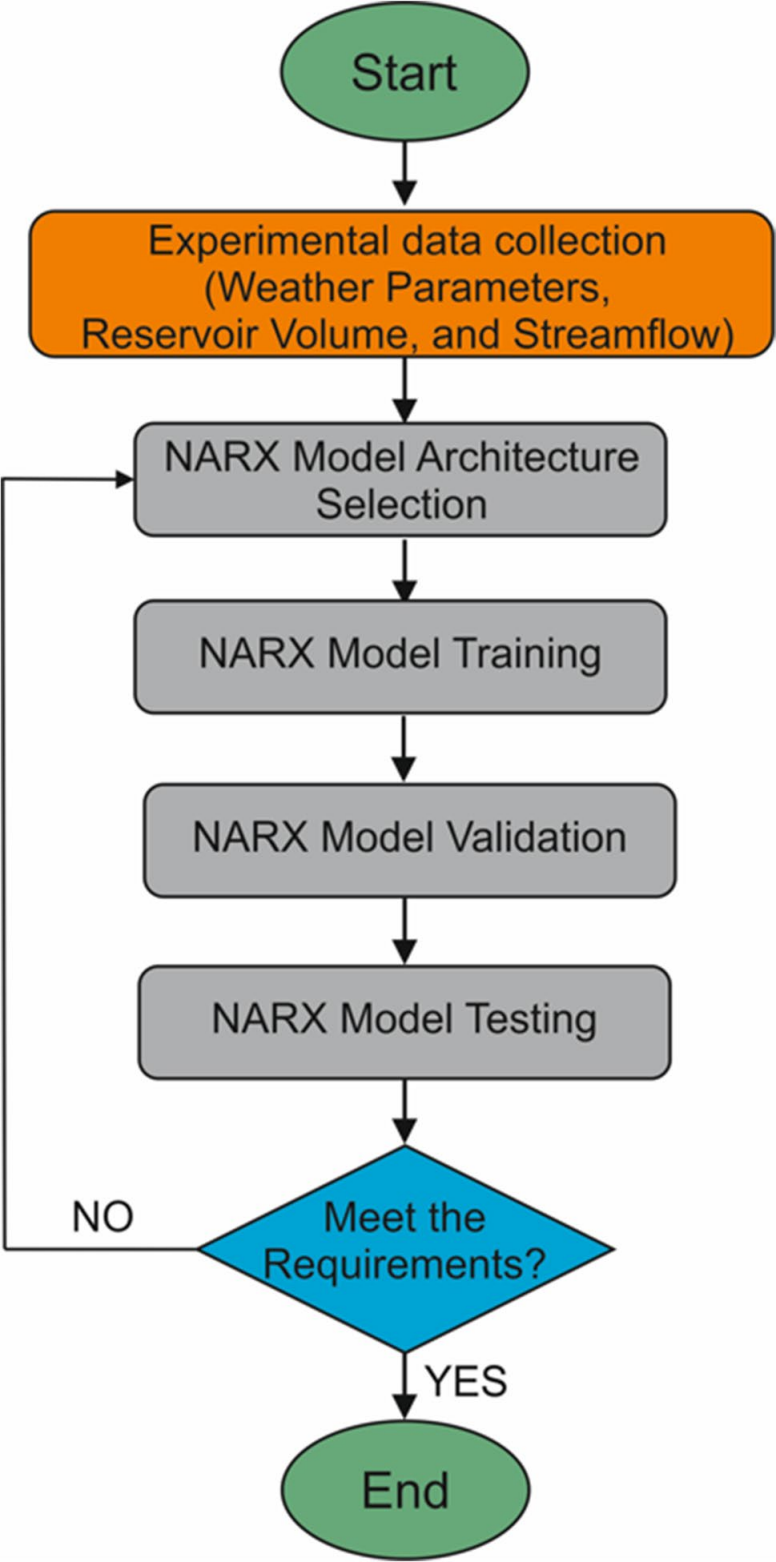
Stages of NARX application

SWAT and NARX were applied for predicting reservoir volumes and streamflow in the Tersakan Basin. Flowcharts representing the key stages of SWAT and NARX models are presented in Figs. 1 and 2, respectively. Below, we first provide background information about our study area. Then, we provide details for SWAT and NARX applications.

### Study area

**Fig. 3:**
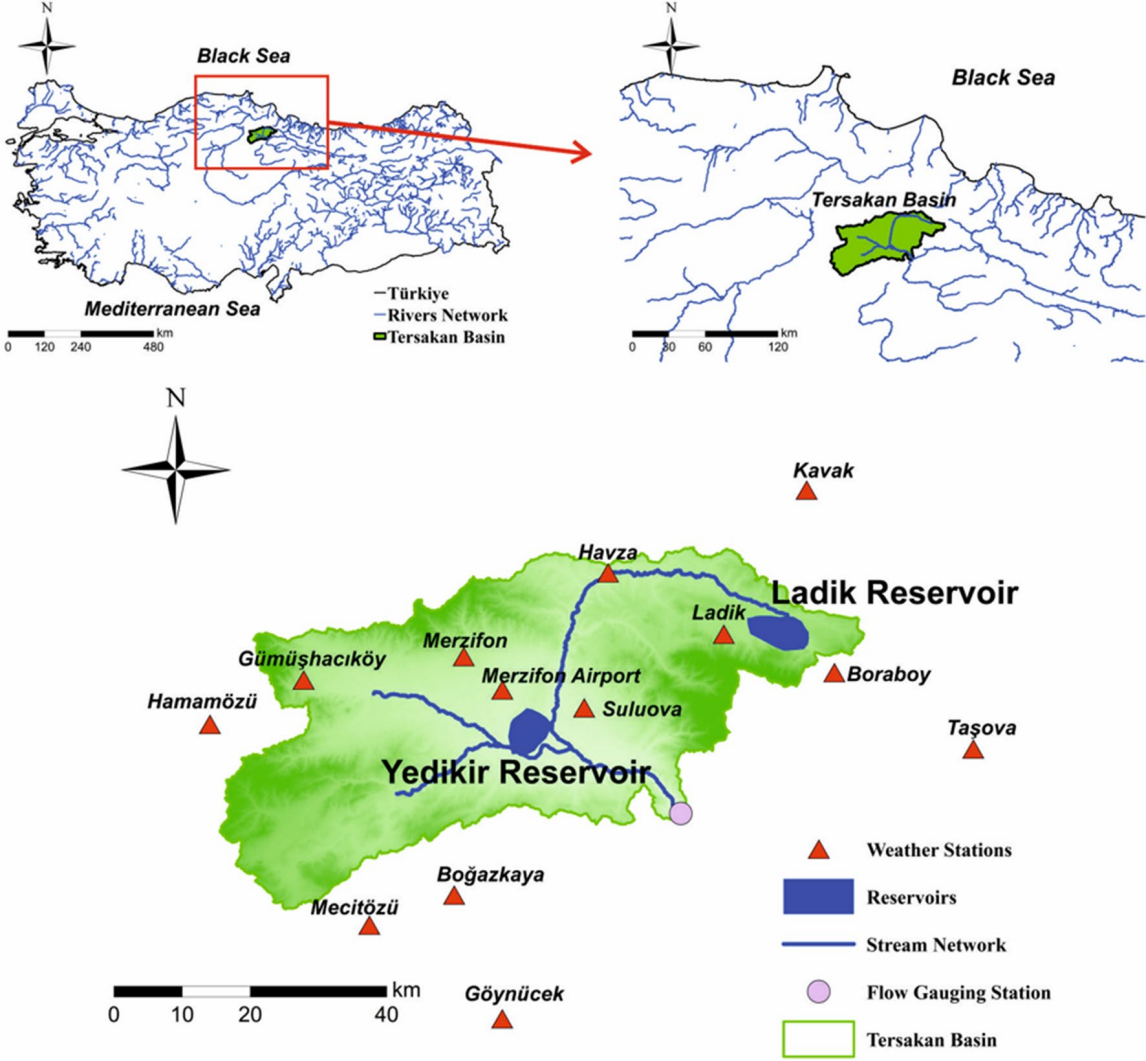
Location of Tersakan Basin, weather station, stream network

Tersakan Basin is located to the north-central Türkiye (Fig. 3). The Tersakan Stream starts from Ladik Reservoir located to the east of the basin. Tersakan Stream irrigates Merzifon (located to the north) and Suluova (located at the center) districts. Due to large agricultural areas covering about 88 km^2^ in Suluova, the streamflow is significantly lower at the basin outlet (Anonymous 2019). The length of the Tersakan Stream is about 100 km and annual flow is 0.125 × 10^9^ m^3^. Maximum, minimum, and average flows are 317 m^3^/s, 0.021 m^3^/s, and 3.96 m^3^/s, respectively.

Tersakan Basin’s total area is 2206 km^2^. Ladik Reservoir was created by State Hydraulic Works for irrigation purposes in 1973 by constructing a regulator at the outlet of Ladik Lake. Tersakan Stream starts from this location (Tübitak Marmara Research Center 2010). Ladik Reservoir’s volume and surface area are 4854 × 10^4^ m^3^ and 13.3 km^2^, respectively. Yedikir Reservoir was built between 1982 and 1985 and provides irrigation service to approximately 74 km^2^ of area. Yedikir Reservoir’s surface area is 5.93 km^2^ and its volume is 5710 × 10^4^ m^3^.

**Fig. 4:**
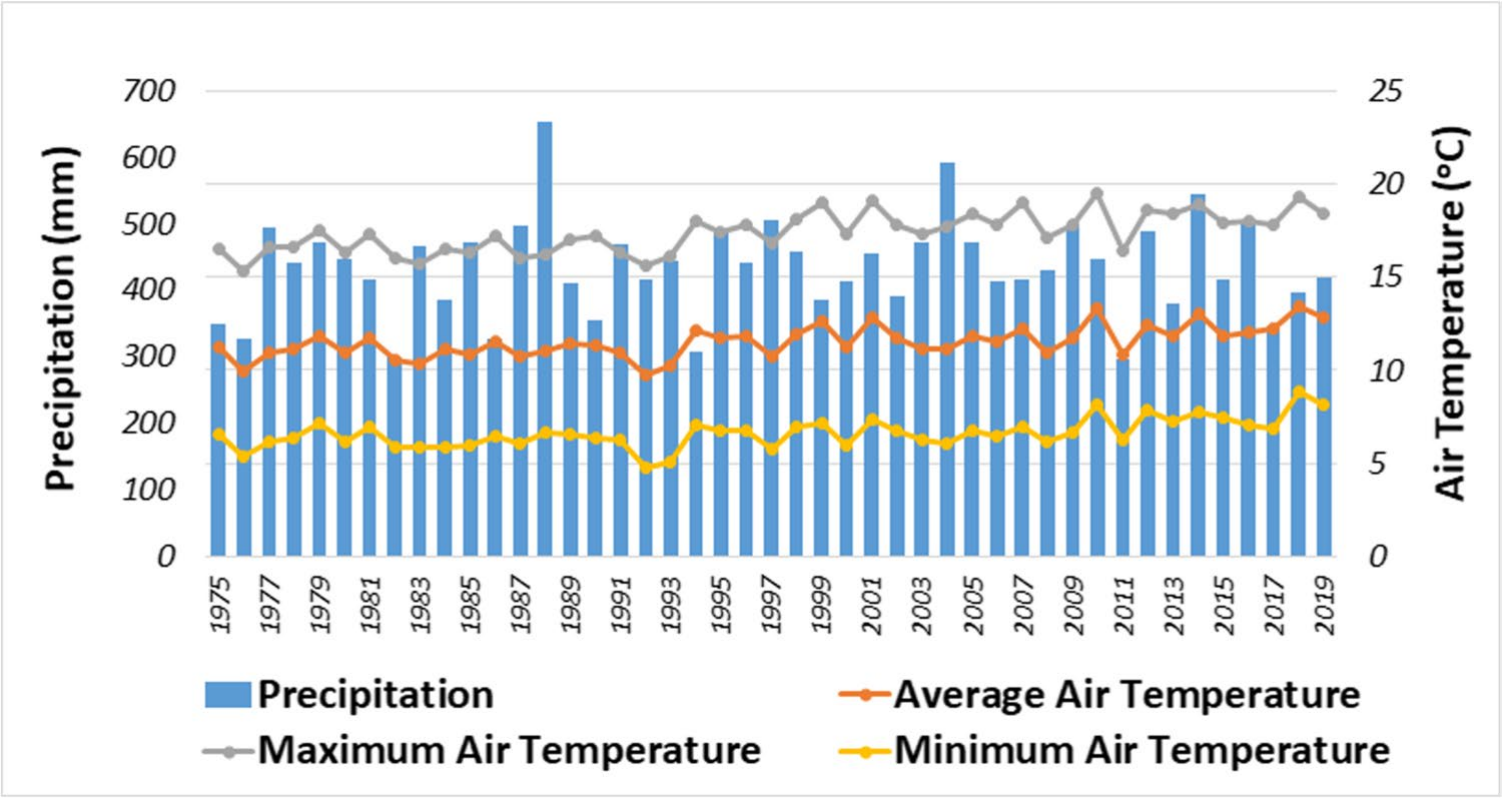
Annual minimum, maximum, and average air temperature and total precipitation

**Fig. 5:**
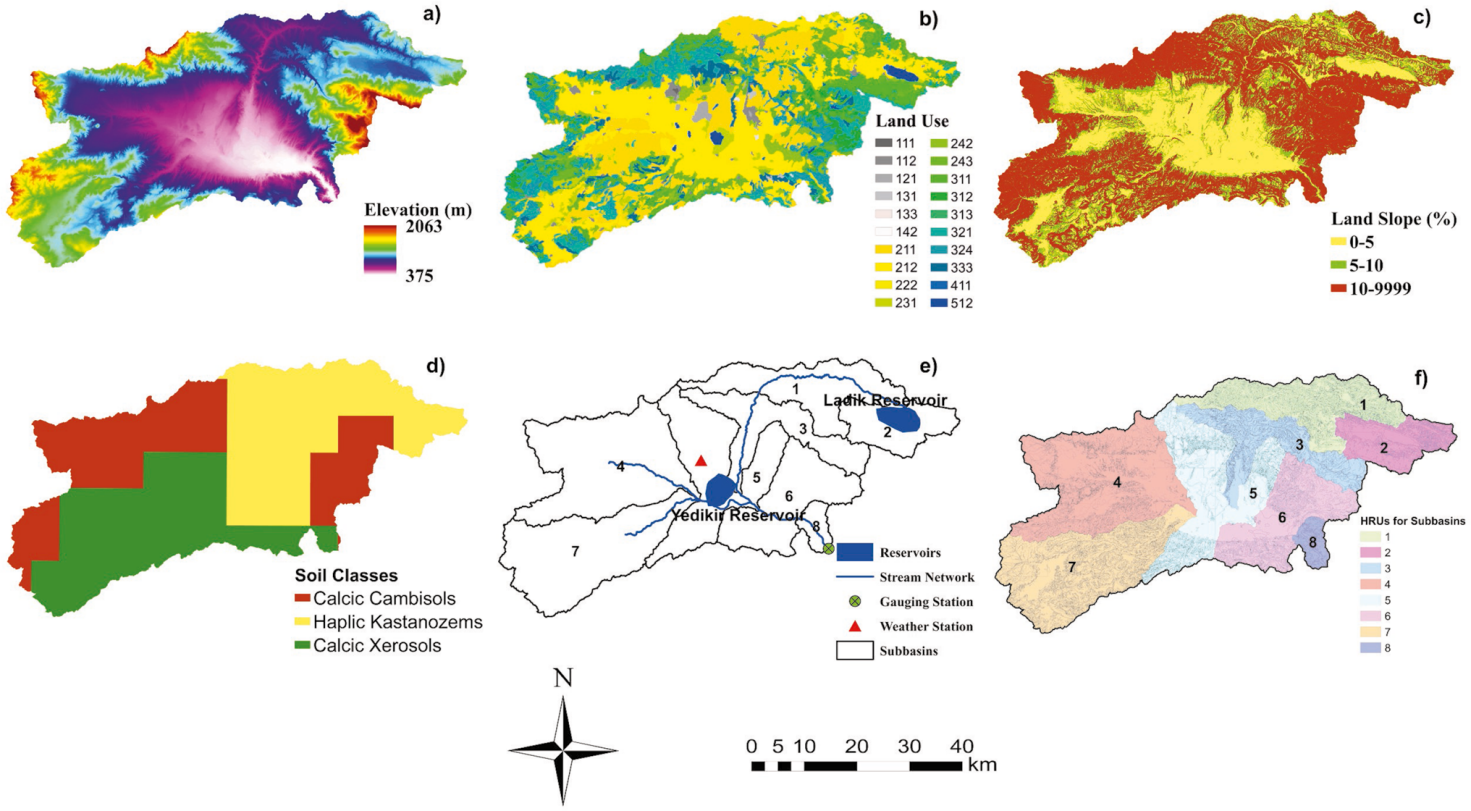
Spatial input data (**a**) DEM, (**b**) land use cover, (**c**) slope, (**d**) soils, (**e**) sub-basins, (**f**) HRUs

Although there were multiple meteorological stations within the basin (Fig. 3), only a single meteorological station had regular data records for long time periods. Figure 4 shows precipitation and minimum, maximum, and average air temperatures measured at this station from 1975 to 2019. The annual average precipitation during the 1975–2019 period was 434 mm and the annual average air temperature was 12°C. The annual minimum and maximum average air temperatures were 7°C and 17°C, respectively. In general, the basin has cold, semi-arid (steppe) climate, as categorized by the Köppen-Geiger climate classification system (Peel et al. 2007). The elevation in the basin ranges from 375 to 2063 m (Fig. 5a).

### Data used

SWAT necessitates a comprehensive dataset including topography, soils, land use/cover, and climatic variables such as minimum and maximum air temperature, precipitation, relative humidity, solar radiation, and wind speed (Fig. 5a, b, d). Additionally, information on land management practices is essential for accurately characterizing watershed processes. Digital elevation model (DEM), soil, and land use/cover data for the Tersakan Basin (Fig. 5) were acquired from global datasets due to unavailability of data from local sources (Table 2). The spatial resolution of these data was quite low (Table 2). Daily meteorological data was available from State Meteorology Service. Streamflow, and reservoir operation data, and data on local agricultural practices were obtained from State Hydraulic Works and local organizations (Table 2).

**Table 2:** Data characteristics and sources

Data	Spatial resolution	Temporal resolution	Data source
Digital elevation model	10 m × 10 m	–	State Hydraulic Works
Soil	1/5.000.000	–	FAO-UNESCO World Soil Map
Land use/cover	30 m × 30 m	–	CORINE 2018
Climate	–	Daily	State Meteorology Service
Reservoir characteristics	–	–	State Hydraulic Works
Reservoir volume	–	Monthly	State Hydraulic Works
Streamflow	Monthly		State Hydraulic Works
Management	–		Local Agricultural Experts

Land use/cover characteristics in the Tersakan Basin is explained based on CORINE 2018 data in Table 3. Corine 2018 dataset has been used to characterize land use/cover in many previous studies (Germeç and Ürker 2023; Llanos-Paez et al. 2023). Tersakan Basin has a high percentage of non-irrigated arable lands with 18.7% coverage and broad-leaved forests with 13.3% coverage. Permanently irrigated area cover 11.5% and land principally occupied by agriculture with significant areas of natural vegetation cover 10%. Natural grasslands and transitional woodland-shrub occupy 11.4% and 13%, respectively. There are three types of soil in the Tersakan Basin. These are calcic cambisols, haplic kastanozems, and calcic xerosols.

**Table 3:** Land use/cover in the Tersakan Basin

Corine 2018 Code	Description	Area (hectare)	Percentage (%)
111	Continuous urban fabric	76	0.03
112	Discontinuous urban fabric	14852	6.73
121	Industrial or commercial units	329	0.15
122	Road and rail networks and associated lands	126	0.06
124	Airports	1042	0.47
131	Mineral extraction facilities	480	0.22
133	Construction sites	219	0.10
142	Sport and leisure facilities	99	0.04
211	Non-irrigated arable land	39363	17.84
212	Permanently irrigated area	24050	10.90
222	Fruit trees and berry plantations	871	0.39
231	Pastures	7664	3.47
242	Complex cultivation patterns	10058	4.56
243	Land principally occupied by agriculture with significant areas of natural vegetation	20938	9.49
311	Broad-leaved forest	27955	12.67
312	Coniferous forest	5661	2.57
313	Mixed forest	3869	1.75
321	Natural grasslands	23935	10.85
324	Transitional woodland-shrub	27345	12.40
333	Sparsely vegetated areas	9226	4.18
411	Inland marshes	224	0.10
512	Water bodies	2203	1.00

For the NARX model, meteorological data consisting of maximum temperature, minimum temperature, precipitation, wind speed, relative humidity, and solar radiation were used as input. The NARX model outputs included water volumes of Ladik and Yedikir Reservoirs and streamflow the basin outlet.

### SWAT model development

SWAT is a semi-distributed, time-continuous, ecohydrological model generally applied at the watershed scale (Arnold and Fohrer 2005) and designed to simulate water, nutrient, and sediment transport (Arnold et al. 1998, 2012; Neitsch et al. 2005). It works with hydrological response units (HRUs) which are areas with unique characteristics identified by land use, soil type, and slope. SWAT runs on a daily time step and can simulate plant growth, water quality, and reservoir operations in addition to sediment and nutrient movement and water balance (Arnold et al. 2012).

The ArcSWAT interface program with revision 664 version of SWAT2012 was used to set up the hydrological model. In the initial phase, the watershed was partitioned into sub-basins using the DEM-based option in SWAT, with an area threshold of 220 km^2^ (approximately 10% of the watershed area) (Fig. 5e). Subsequently, certain sub-basins were merged to ensure that reservoirs are located within single sub-basins. Hydrologic response units (HRUs) were created by combining DEM, soil, land use, and slope maps (Fig. 5f) (Neitsch et al. 2005). A 5% threshold for land use, soil, and slope was applied during generation to ensure that variations in land use, soil, and slope were adequately represented in the model. This approach helped capture the spatial heterogeneity of the landscape while ensuring that computational burdens were minimized by excluding small areas (Arnold et al. 2012). We used the variable storage method for river channel routing method for and the Penman/Monteith method for potential evapotranspiration estimation.

Water volume data obtained from Ladik and Yedikir Reservoirs and streamflow data collected at the basin outlet were used to calibrate and validate the SWAT model. Model calibration aims to find a specific set of model parameters that can accurately capture the behavior of the system. Model calibration is an iterative process, where observed and simulated values are continuously compared using different parameters sets. The process continues until the parameter set that provides the most satisfactory results is determined. In this study, a software package called SWAT-CUP (Abbaspour 2015), which provides automatic model calibration, was used. Although several algoritms are available within the SWAT-CUP, we preferred to use the Sequential Uncertainty Fitting Version 2 (SUFI-2) algorithm, which is the most frequently used algorithm for SWAT model calibration and has been proved to be successful (Aibaidula et al. 2022; Mengistu et al. 2019). For calibration and validation, the procedure explained in Abbaspour et al. (2007) and Abbaspour et al. (2015) was used. Twenty-eight parameters that could affect streamflow, reservoir storage, and irrigation were used for calibration. The parameter set was determined based on the available literature where most used parameters for SWAT calibration were listed (Table 4). For each parameter, the lower and upper limit values were also selected from the literature. We included all 28 parameters in calibration. Manual calibration was conducted prior to automatic calibration with SWAT-CUP. A sensitivity analysis is applied to be able to understand the response of the basin to different parameters. In SWAT-CUP, the SUFI-2 algorithm was run 500 times in each iteration. Iterations were continued until the best fit between the simulated and observed values was reached (Abbaspour et al. 2015). At the end of each iteration, new parameter ranges produced by the algorithm were used. Reservoirs are important elements that greatly affect the hydrological dynamics in the SWAT model (Phiri et al. 2021). Irrigation activities also affect the water movement greatly. For this purpose, the parameters that can affect reservoir volumes and irrigation water use were determined and calibration/validation process was carried out for Ladik and Yedikir Reservoirs.

**Table 4:** Parameters selected for streamflow and reservoir volume calibration, calibration ranges, and type of calibration (*v* means that the existing parameter value is replaced by a given value and r means that the existing parameter value is multiplied by (1 + a given value (Abbaspour 2015))

Parameters	Definition	Lower and upper limits used for calibration of sub-basins 1 and 2	Lower and upper limits used for calibration of sub-basins 3, 4, 5, and 7	Lower and upper limits used for calibration of sub-basins 6 and 8	Calibration value for lad sub-basins 1 and 2	Calibration value for sub-basins 3, 4, 5, and 7	Calibration value for sub-basins 6 and 8
r_CN2.mgt	Curve number for moisture condition	− 0.1–0.1	− 0.2–0.15	0–0.1	− 0.154889	0.033084	0.059101
v_ALPHA_BF.gw	Baseflow alpha factor	0–1	0–1	0–1	0.760899	0.689004	0.081291
v_GW_DELAY.gw	Groundwater delay	0–50	0–500	0–500	8.182090	405.34610	384.58044
v_GWQMN.gw	Threshold depth of water in the shallow aquifer required for return flow to occur	100–700	0–3000	0–5000	108.71205	2267.8537	1456.8583
v_CH_N2.rte	Manning’s “*n*” value for the main channel	0–0.3	− 0.01–0.3	− 0.01–0.3	0.082613	0.224540	0.166480
v_CH_K2.rte	Effective hydraulic conductivity in main channel alluvium	0–10	− 0.01–500	− 0.01–500	2.588405	192.03659	46.910172
v_GW_REVAP.gw	Groundwater “revap” coefficient	0–0.1	0.02–0.2	0.02–0.2	0.036001	0.050834	0.094206
v_REVAPMN.gw	Threshold depth of water in the shallow aquifer for “revap” to occur	0–500	0–800	0–1000	278.60247	403.13742	212.44860
r_SOL_AWC(1).sol	Available water capacity of the soil layer	− 0.5–0.6	− 0.5–0.8	− 0.8–0.8	0.369330	0.007883	0.749207
r_SOL_BD(1).sol	Moist bulk density	− 0.5–06	− 0.5–0.6	− 0.2–0.65	− 0.187008	0.059004	0.266672
v_SFTMP.bsn	Snowfall temperature	− 5–5	–	–	− 1.539491	–	–
v_OV_N.hru	Manning’s “*n*” value for overland flow	0.01–1.0	0.01–1	0.01–30	0.903051	18.300280	17.205547
r_SLSUBBSN.hru	Average slope length	− 0.1–0.2	− 0.2–0.6	0–0.1	− 0.085284	0.123350	0.035402
v_ESCO.hru	Soil evaporation compensation factor	0–1	0–1	0–1	0.941368	0.273712	0.994636
r_SOL_K(1).sol	Saturated hydraulic conductivity	− 0.8–0.8	− 0.8–0.8	− 0.8–0.8	− 0.137758	− 0.625972	0.253495
v_RCHRG_DP.gw	Deep aquifer percolation fraction	0–1	0–1	0–1	0.027365	0.648672	0.712874
v_GWHT.gw	Initial groundwater height	0–25	0–25	0–25	10.981455	6.026505	23.453829
r_SURLAG.bsn	Surface runoff lag time	− 0.5–0.6	–	–	–	–	–
v_EPCO.hru	Plant uptake compensation factor	0–1	0–1	0–1	0.775981	0.277203	0.416822
v_SNOCOVMX.bsn	Minimum snow water content that corresponds to 100% snow cover	0–50	–	–	66.418297	–	–
v_SMTMP.bsn	Snow melt base temperature	− 5–5	–	–	− 0.473332	–	–
v_SMFMX.bsn	Maximum melt rate for snow during year (occurs on summer solstice)	0–10	–	–	1.954915	–	–
v_TIMP.bsn	Snow pack temperature lag factor	0–1	–	–	0.705938	–	–
v_SMFMN.bsn	Minimum melt rate for snow during the year (occurs on winter solstice)	0–10	–	–	2.188665	–	–
v_EVRSV.res	Lake evaporation coefficient	0–1	0–1	–	0.527400	0.524337	–
v_RES_K.res	Hydraulic conductivity of the reservoir bottom (mm/hr)	0–1	0–1	–	0.024410	0.192192	–
v_IRR_EFF.mgt	Irrigation efficiency	0–1	0–1	–	0.983732	0.558905	–
v_AUTO_WSTRS.mgt	Plant water stress factor	0–1	0–1	–	0.342537	0.041917	–

We ran the SWAT model for the 2000–2017 period, where the first 5 years were used for model warm-up. The warm-up period refers to the initial period of the simulation during which the model adjusts to initial conditions, for various variables such as soil moisture content and reservoir volumes, to reach a stable state before actual model simulations. The duration of the warm-up period can change depending on the characteristics and complexity of the watershed being modeled, and spatial and temporal resolution of the input data used (Prasad et al. 2015). Typically, a warm-up period of 2–10 years is recommended for most SWAT model simulations; however, much longer warmup periods has also been used (Prasad et al. 2015; Schuol et al. 2008; Wang and Kalin 2011; Wu et al. 2012). We used a warm-up period of 5 years, to let the model adjust initial conditions especially for reservoirs.

During model calibration, we followed a multi-variable and multi-site calibration approach. This approach involved adjusting multiple model parameters simultaneously and across multiple sites. This approach has been applied before in basins where spatial variations are high and proved to be more successful in simulating hydrological processes (Cao et al. 2006; Moussa et al. 2007; Shresthaa et al. 2016)*.* In the SWAT model calibration, water volumes at the Ladik Reservoir were used to calibrate the SWAT model for sub-basins 1 and 2 and water volumes at the Yedikir Reservoir were used to calibrate the SWAT model for sub-basins 3, 4, 5, and 7. The streamflow at the basin outlet was used to calibrate the SWAT model for sub-basins 6 and 8. The parameters related to the basin (.bsn) were selected in the calibration based on Ladik Reservoir volumes only and the results found here were added to the SWAT model for all sub-basins. Water volumes at the Ladik Reservoir were available for the 2010–2017 period, where the data from 2010 to 2014 (60 months) were used for calibration and 2015–2017 (36 months) for validation. For Yedikir Reservoir, water volume data could be reached for the 2010–2016 period. Here, we used the 2010–2014 (60 months) period for calibration and the 2015–2016 (24 months) period for validation. Streamflow at the basin outlet was available only for the 2013–2017 period. The 2013–2015 (36 months) and 2016–2017 (24 months) periods were selected for calibration and validation, respectively.

### NARX model development

**Fig. 6:**
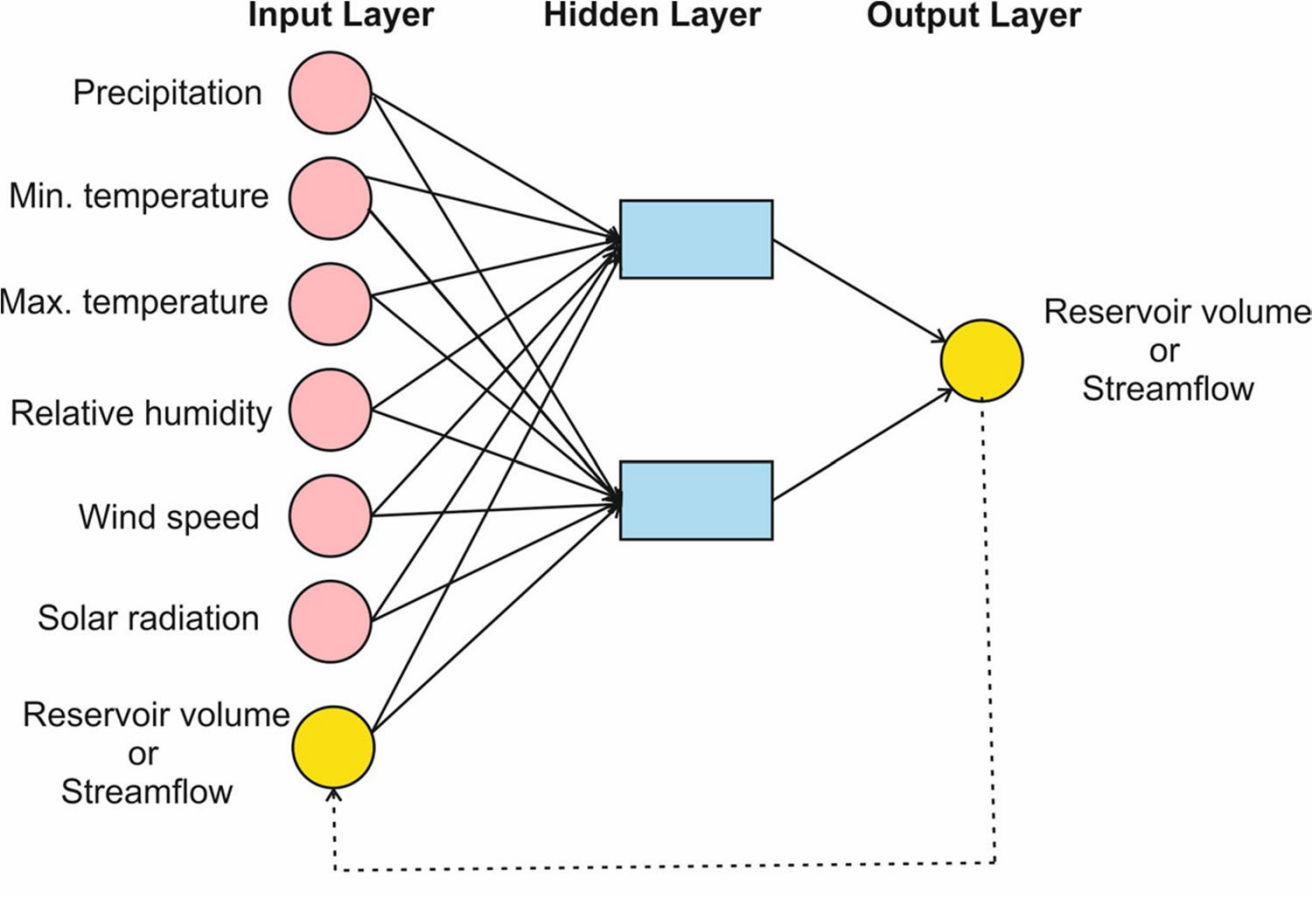
Architecture of the NARX Model

NARX is a type of RNN that deals with forecasting time series data (Chang et al. 2013, 2022; Lin et al. 1998; Menezes and Barreto 2008; Wunsch et al. 2018). The NARX model has three different architectures (Shen and Chang 2013). A statistical neural network is the first type of architecture. In this type, the NARX models use the target as an input during model training and model testing. The second type has a serial, parallel configuration, in which the target is used as an input in during model training, and the output value is feedback as an input value during model testing. Usually, the NARX model performance was stronger during the training phase, but weaker during model testing. The last type is a parallel configuration. In this type, the output value is used as an input during the testing and training phases. This study used a parallel configuration because this type leads to a strong fault tolerance (Chang et al. 2022). Figure 6 shows the NARX model architecture used in this study. The NARX model consisted of input, hidden, and output layers. The outputs create new inputs and the inputs can delay for a certain time steps. Equation 1 can be used for forecasting N-step-ahead (*N* ≥ 1):

1 $$z \left(t+N\right)=f[z\left(t+N-1\right),\dots ,z\left(t+N-q\right); \;U(t)]$$

In Eq. (1), $f(.)$ is the nonlinear function. $z \left(t+N\right)$ and $U(t)$ output value and denote the input vector at the *t* time step, respectively. $q$ is the order of output memory. $z\left(t+N-q\right)$ and $U(t)$ are input regressors. The $z\left(t+N-i\right)$ regressor (*i* is 1 to q) acts as the autoregressive model in the time series, and another regressor $U(t)$ also acts as an implicit exogenous variable.

In this study, the NARX model was used to estimate the water volumes at the Ladik and Yedikir Reservoirs and streamflow at the basin outlet. The NARX model network was trained using the Scaled Conjugate Gradient algorithm (Møller 1993) and the transfer functions of layers were selected as the sigmoid type. In this algorithm, a feedforward ANN architecture is used, where connection weights of neurons are optimized at the same time (Chen and Chang 2009). In practice, the scaled conjugate algorithm was found to be more effective than the classical backpropagation algorithm (Chiang et al. 2004). Model construction and application were undertaken on the MATLAB 2016a software. We used the default learning rate (0.01) in all simulations.

The number of hidden neurons and delays were determined based on the least complex model structure that might produce adequate results based on the available literature (Alsumaiei 2020; Chiang et al. 2004; Wunsch et al. 2018; Yang et al. 2019). First, we tested the number of neurons and delay parameters for various values. This analysis revealed that the network structures with 1–10 hidden neurons and 1–10 delay numbers provided better model performance. Delay numbers of NARX model can help reduce the sensitivity of the network system (Li et al. 2017). For selection of the optimum parameter values and further improvement, the NARX model was run several times by selecting values from this range. The optimum parameter values were selected as those that provided the highest performance.

Monthly averages (for minimum and maximum temperature, relative humidity, and solar radiation) or totals (for precipitation) were calculated for climatic variables and used as input. All data were normalized to be between 0 and 1 (Eq. 2). The data were randomly divided into three sets: training, validation, and testing sets. Seventy percent of the data was used for training and 15% for validation and 15% for testing.

2 $$X=\frac{{X}_{ori}-{X}_{min}}{{X}_{max}-{X}_{min}}$$

In Eq. 2, ${X}_{\text{min}}$, ${X}_{\text{max}}$, ${X}_{\text{ori}}$, and $X$ were minimum, maximum, original, and normalized values, respectively.

### Performance evaluation and comparison

We used five performance measures to evaluate the success in model calibration and validation. These were coefficient of determination (*R*^2^) (Krause et al. 2005), Nash–Sutcliffe Efficiency (NSE) (Nash and Sutcliffe 1970), root-mean-square error (RMSE), normalized root mean squared error (NRMSE) (Armstrong and Collopy 1992), and Kling-Gupta efficiency (KGE) (Gupta et al. 2009).

In Eqs. 3–6, ${E}_{\text{observed}}$shows the observed value, and ${E}_{\text{predicted}}$ shows the predicted one. ${\overline{E} }_{\text{predicted}/\text{observed}}$ is the mean of the predicted/observed values, ${E}_{\text{predicted}/\text{observed}}^{t}$ is the predicted/observed values at time *t*, and ${E}_{\text{predicted}\_\text{average}}$ is the average of the predicted values. *N* is the number of data.

*R*^2^ (Eq. 3) range from 1 to 0, and when it is close to 1 there is a perfect relationship between the actual and predicted values (Krause et al. 2005; Yang et al. 2017).

3 $${R}^{2}={\left\{\frac{{\sum }_{i=1}^{N}\left({E}_{Observed}^{t}-{\overline{\text{E}} }_{\text{Observed}}\right)\left({\text{E}}_{predicted}^{t}-{\overline{E} }_{predicted}\right)}{\sqrt{{\sum }_{i=1}^{N}{\left({E}_{Observed}^{t}-{\overline{\text{E}} }_{\text{Observed}}\right)}^{2}}\sqrt{{\sum }_{i=1}^{N}{\left({\text{E}}_{predicted}^{t}-{\overline{E} }_{predicted}\right)}^{2}}}\right\}}^{2}$$

NSE (Eq. 4) changes between − ∞ and 1 and the values close to 1 denote better performance.

4 $$NSE=1-\frac{{\sum }_{i=1}^{N}{({E}_{observed}-{E}_{predicted})}^{2}}{{\sum }_{i=1}^{N}{({E}_{predicted}-{E}_{predicted\_average})}^{2}}$$

The RMSE (Eq. 5) presents the error between the simulated and observed values, and this value is a widely used error index statistic (Singh et al. 2005).

5 $$\text{RMSE}=\sqrt{\frac{\sum_{i=0}^{N}{({E}_{predicted}-{E}_{observed})}^{2}}{N}}$$

Model performances for SWAT and NARX were also evaluated based on NRMSE (Eq. 6), the relative form of RMSE, which provided a better comparison by normalizing the volumes of reservoirs with different capacities by the mean values.

6 $$NRMSE (\%)=\frac{\sqrt{\frac{\sum_{i=0}^{N}{({E}_{predicted}-{E}_{observed})}^{2}}{N}}}{{\overline{E} }_{observed}}\times 100$$

KGE metric (Eq. 7) ranges from − ∞ and 1 (Pham et al. 2021). The closer the model result is to 1, the more perfect the model performance.

7 $$KGE=1-\sqrt{{\left(r-1\right)}^{2}+{\left(\propto -1\right)}^{2}+{\left(\beta -1\right)}^{2}}$$

In Eq. 7, ∝ is a relative variability in the simulated and observed values. *r* is the Pearson coefficient and β represents the bias:

8 $$\propto =\frac{{\sigma }_{\widehat{y}}}{{\sigma }_{y}} \text{and }\beta =\frac{{\mu }_{\widehat{y}}}{{\mu }_{y}}$$

where ${\sigma }_{y}$ is the standard deviation of the simulating values, and ${\sigma }_{\widehat{y}}$ is the standard deviation of the observations. ${\mu }_{\widehat{y}}$ and ${\mu }_{y}$ are simulating and observation mean, respectively.

More than one metric should be taken into account when evaluating models as individual metrics may have weaknesses (Bennett et al. 2013). In this study, we used five metrics for comparison of model performance: *R*^2^, RMSE, NRMSE, NSE, and KGE. RMSE and *R*^2^ are among the metrics chosen for model performance due to their wide usage areas. NSE is a parameter that is sensitive to peaks and may provide a more reliable assessment. KGE is a relatively new metric developed based on NSE (Pham et al. 2021). It is very popular in hydrological models by addressing the shortcomings in NSE by incorporating bias and variance terms (Akbarian et al. 2023). Since two reservoirs of different capacities were compared in the study, it would be useful to express the results in relative terms scaled to mean values. Therefore, the NRMSE metric was used.

## Results and discussion

### Performance of the SWAT model

**Fig. 7:**
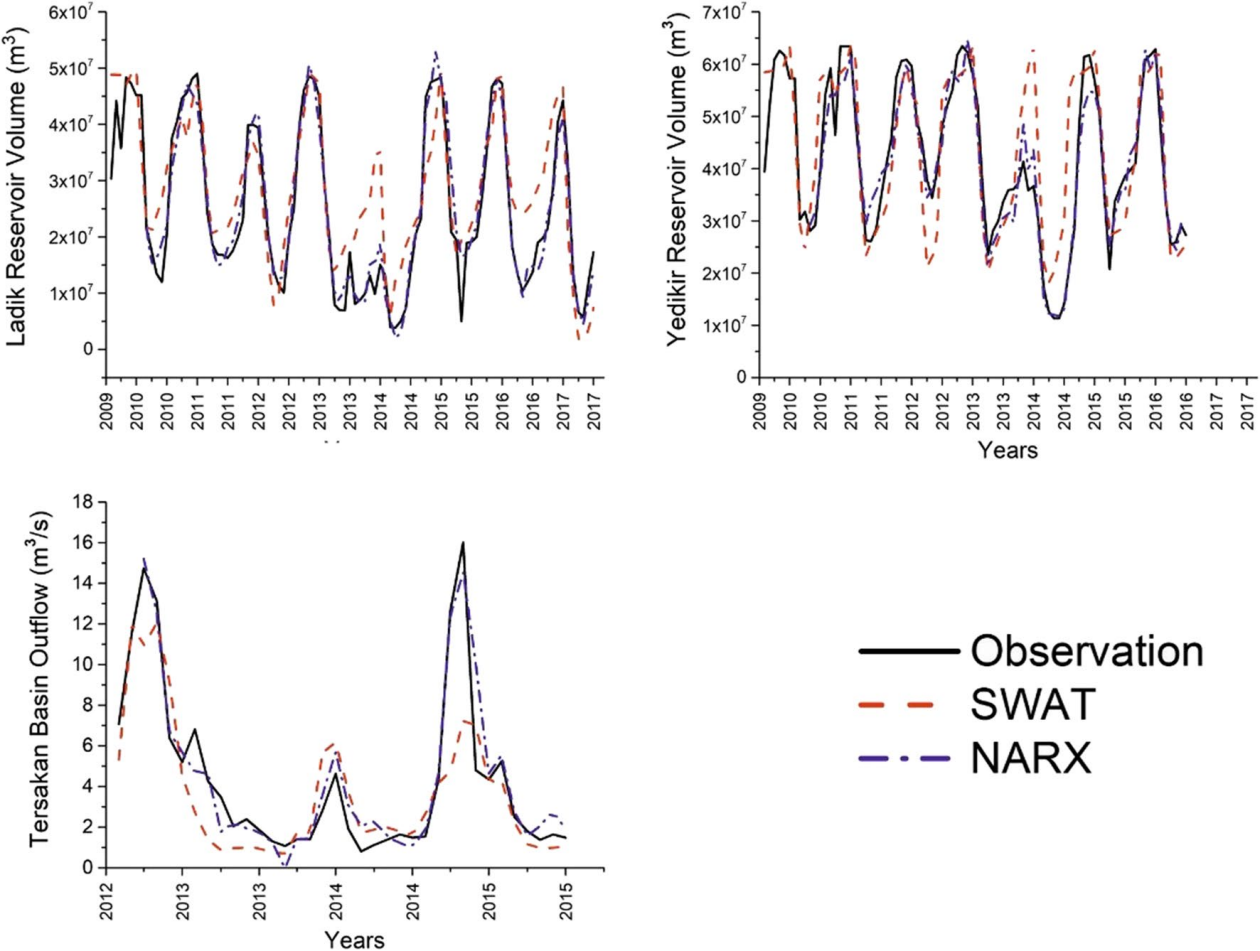
The comparison of the SWAT and NARX models’ predicted and observed results for reservoirs volume and streamflow

The SWAT model of the Tersakan Basin included 8 sub-basins (Fig. 5e) and 220 HRUs (Fig. 5f). Sub-basins are spatially related to each other and have a geographical location within the basin. The sub-basin boundary is obtained in such a way that the entire area within any sub-basin flows to the outlet of the other sub-basin (Arnold et al. 2012). The parameters used in model calibration, their descriptions, initially selected ranges, and calibration outputs are shown in Table 4. Table 5 and Fig. 7 present model calibration and validation results.

**Table 5:** Summary of SWAT Model statistical results for Ladik and Yedikir reservoirs volume and streamflow

Variable	Calibration					Validation				
	NSE	*R*^2^	RMSE	NRMSE	KGE	NSE	*R*^2^	RMSE	NRMSE	KGE
Water volume at the Ladik Reservoir	0.69	0.76	8.3 × 10^6^ m^3^	33%	0.73	0.64	0.67	8.3 × 10^6^ m^3^	32%	0.79
Water volume at the Yedikir Reservoir	0.65	0.69	8.9 × 10^6^ m^3^	21%	0.83	0.41	0.56	10.7 × 10^6^ m^3^	26%	0.73
Streamflow at the basin outlet	0.63	0.66	2.5 m^3^/s	57%	0.67	0.22	0.79	3.7 m^3^/s	71%	0.29

The sensitivity analysis showed that reservoir volumes were most sensitive to GW_Delay.gw (delay time for aquifer recharge), RCHRG_DP.gw (aquifer percolation coefficient), and CH_K(2).rte (effective hydraulic conductivity of channel), and streamflow was most sensitive to SURLAG.bsn (surface runoff lag coefficient), CH_K(2).rte (Effective hydraulic conductivity of channel), and GW_REVAP.gw (Revap coefficient).

The *R*^2^, NSE, KGE, RMSE, and NRMSE values calculated between the observed and predicted water volumes at the Ladik Reservoir were 0.76, 0.69, 0.73, 8.3 × 10^6^ m^3^, and 33% respectively, for the calibration period and 0.67, 0.64, 0.79, 8.3 × 10^6^ m^3^, and 32% respectively for the validation period. Based on observed and predicted water volumes at the Yedikir Reservoir, *R*^2^, NSE, KGE, RMSE, and NRMSE values were calculated as 0.69, 0.65, 0.83, 8.9 × 10^6^ m^3^, and 21% respectively, for the calibration period and 0.56, 0.41, 0.73, 10.7 × 10^6^ m^3^, and 26% respectively for the validation period. Moriasi et al. (2015) state that the SWAT model performance can be classified as “satisfactory” when 0.5 < NSE < 0.7 and 0.6 < *R*^2^ < 0.75 for flow predictions at the daily, monthly, and annual scales. Model performance was proposed to be “good” when 0.7 < NSE < 0.80 and 0.75 < *R*^2^ < 0.85 and “very good” when NSE > 0.80 and *R*^2^ > 0.85. KGE model performance can be divided into three groups, “poor performance” (0.5 > KGE > 0), “intermediate” (0.75 > KGE > 0.5), and “good performance” (KGE > 0.75) (Moriasi et al. 2015). They did not identify criteria based on RMSE, as the values for RMSE could change based on the units of the variable. However, Yuzer and Bozkurt (2022) mentioned that the SWAT model performance is excellent when NRMSE < 10%. Based on these criteria, the model performance was found to be “satisfactory” based on NSE for the calibration and validation periods for predicting water volumes at the Ladik Reservoir. Based on *R*^2^, it was “good” during model calibration and “satisfactory” during model validation. Based on KGE, it was “intermediate” during model calibration and “good” during model validation. For prediction of water volumes at the Yedikir Reservoir, model performance was found to be “satisfactory” based on NSE, and *R*^2^ during model calibration, but “unsatisfactory” during validation. Based on KGE, it was “good” during model calibration and “intermediate” during model validation. The NRMSE values were higher than 10% in all cases. Here, we should mention that most of the SWAT model evaluation criteria were developed based on flow estimations. No specific criteria are available for models that simulate reservoir volumes. Predicting reservoir volumes is more challenging due to the high variability of water inflows and outflows. A few studies are available in the literature that evaluated model performance for water volume prediction in lakes and reservoirs. Beharry et al. (2021) showed that the NSE value between observed and predicted reservoir volumes were 0.67 during the calibration period and 0.70 during the validation period. Kim and Parajuli (2012) modeled the reservoir outflow option in SWAT. Considering the SWAT model performances, they found that the NSE value was 0.60 in the calibration period and 0.62 in the validation period. NSE and *R*^2^ values calculated between predicted and observed reservoir volumes were found to be 0.36–0.60 and 0.54–0.75 during model calibration and 0.23–0.13 and 0.49–0.50 during model validation for two irrigation reservoirs in Türkiye, respectively (Jouma and Dadaser‐Celik 2022). These results suggest that the SWAT model performance obtained for predicting water volumes at the Ladik and Yedikir Reservoirs were compatible with the available literature.

Based on the observed and predicted streamflow at the basin outlet, the *R*^2^, NSE, KGE, RMSE, and NRMSE values were calculated as 0.66, 0.63, 0.67, 2.5 m^3^/s, and 57%, respectively, for the calibration period and 0.79, 0.22, 0.29, 3.7 m^3^/s, and 71%, respectively, for the validation period. Calibration period results showed that model performance was “satisfactory” based on NSE and *R*^2^ criteria proposed by Moriasi et al. (2015). For the validation period, *R*^2^ result showed that model performance was “good,” but NSE result showed that it was “unsatisfactory.” This study showed that there is a strong relationship in calibration period between predicted and measured values. But during the validation of the SWAT model for streamflow, the degree of relationship between observed and predicted values were lower. Tan et al. (2019) provided a review of SWAT model performance in Southeast Asia for monthly and daily streamflow prediction based on *R*^2^ and NSE values. More than 60% of the 217 studies performed “very good” for monthly streamflow. However, some other studies provided lower performance due to uncertainties in input data. They also reported that in general, the results of the calibration provided better results than validation.

As can be seen from Table 5, the SWAT model generally provided good to satisfactory results particularly based on NSE value during calibration, but it was sometimes lower during validation. The performance of the SWAT model could be affected by a variety of factors: (1) the SWAT model for the Tersakan Basin was developed with DEM, soil, and land use/cover data obtained from global datasets with low spatial resolutions. Also meteorological data were available only from a single station. Many previous studies showed that SWAT model performance could be affected by the characteristics of meteorological data and DEM, soil, and land use/cover datasets used (Bouslihim et al. 2019; Cuceoglu et al. 2021). In this study, data availability created major challenges (2). Tersakan Basin is a basin, where the hydrologic regime was modified significantly due to construction of reservoirs and irrigation water use in the basin. Desta and Lemma (2017) evaluated the SWAT model results for the Ziway Lake, which is under intense human influence, and stated that the human interventions negatively affected the hydrological results. Similarly, Jouma and Dadaser‐Celik (2022) showed that model performance was lower due to modification of the hydrologic regime in the Develi Basin (Türkiye) (3). The lack of information about irrigation practices in the Tersakan Basin posed another challenge during model calibration. Due to the unavailability of data, we estimated irrigation with the auto-irrigation tool available in SWAT, where irrigation water requirements could be predicted based on crop water stress. We identified the parameters used for estimating water stress during calibration. Considering the high spatial and temporal variability of cropping and irrigation practices across the basin, auto-irrigation tool only provided rough estimates of irrigation water use. Chen et al. (2018) and Chen et al. (2020) also mentioned that auto-irrigation tool available in SWAT could pose some uncertainties on model results.

### Performance of the NARX model

NARX model was developed to predict reservoirs volumes at the Ladik and Yedikir Reservoirs and streamflow at the basin outlet. Delay numbers for the best models were determined to be 7, 9, and 2 for predicting water volumes at the Ladik Reservoir, water volumes at the Yedikir reservoir, and streamflow at the basin outlet, respectively, and the number of neurons for the same variables were 10, 9, and 6, respectively. Model results are shown in Fig. 7 and Table 6.

**Table 6:** Summary of NARX model statistical results for Ladik and Yedikir reservoirs volume and streamflow

Variable	Training					Validation					Testing				
	NSE	R^2^	RMSE	NRMSE	KGE	NSE	R^2^	RMSE	NRMSE	KGE	NSE	R^2^	RMSE	NRMSE	KGE
Water volume at the Ladik Reservoir	0.95	0.96	3.1 × 10^6^ m^3^	13%	0.96	0.93	0.94	3.6 × 10^6^ m^3^	14%	0.90	0.95	0.96	2.6 × 10^6^ m^3^	13%	0.92
Water volume at the Yedikir Reservoir	0.93	0.93	4.1 × 10^6^ m^3^	9.7%	0.92	0.96	0.96	2.6 × 10^6^ m^3^	6%	0.98	0.90	0.93	3.5 × 10^6^ m^3^	16%	0.91
Streamflow at the basin outlet	0.89	0.89	1.5 m^3^/s	32%	0.89	0.95	0.96	0.9 m^3^/s	21%	0.89	0.71	0.79	0.9 m^3^/s	22%	0.81

The *R*^2^, NSE, KGE, RMSE, and NRMSE values calculated between the observed and predicted water volumes measured at the Ladik Reservoir were 0.96, 0.95, 0.96, 3.1 × 10^6^ m^3^, and 13%, respectively, for the training period; 0.94, 0.93, 0.90, 3.6 × 10^6^ m^3^, and 14% for the validation periods; and 0.96, 0.95, 0.92, 2.6 × 10^6^ m^3^, and 22%, for the testing period, respectively. For the Yedikir Reservoir, the *R*^2^, NSE, KGE, RMSE, and NRMSE values between predicted and observed water volumes were 0.93, 0.93, 0.92, 4.1 × 10^6^ m^3^, and 9.7% for the training period; 0.96, 0.96, 0.98, 2.6 × 10^6^ m^3^, and 6% for the validation period; and 0.93, 0.90, 0.91, 3.5 × 10^6^ m^3^, and 16% for the testing period, respectively. Based on model performance criteria, the NARX model performance was found to be “very good” based on NSE and *R*^2^ for all periods for simulating water volumes at the Ladik Reservoir. For prediction of water volumes at the Yedikir Reservoir, model performance was found to be “very good” based on NSE, and *R*^2^ during model training, validation and testing periods. Based on KGE, it was “good” during model training, validation, and testing for Ladik and Yedikir reservoir. Moreover, as the NRMSE value is less than 10%, the NARX model performance is excellent for training and validation periods based on these criteria.

The *R*^2^, NSE, KGE, RMSE, and NRMSE values calculated between predicted and observed streamflow at the basin outlet were 0.89, 0.89, 0.89, 1.5 m^3^/s, and 0.32% for the training period; 0.96, 0.95, 0.89, 0.9 m^3^/s, and 0.21% for the validation period; and 0.79, 0.71, 0.81, 0.9 m^3^/s, and 0.22% for the testing period, respectively. For streamflow, predicted model performance was found to be “very good” based on NSE, and *R*^2^ during model training and validation periods.

For the testing period, the NARX model performance was “good.” Based on KGE it was “good” during model training, validation, and testing period.

There are only a few studies in the literature regarding reservoirs, and in those studies the reservoir inlet or outlet flow was predicted rather than reservoir volumes. Ghazali et al. (2018) studied model performance for simulating monthly reservoir inflow and the *R*^2^ results ranged from 0.73 to 0.90. Yang et al. (2019) also examined the reservoir inflow prediction and they found the NSE result as 0.85. These results showed that in this study results found were similar and compatible with the literature. Results also showed that the NARX model is suitable for reservoir volume prediction.

The NARX model does not use information or data regarding the physical characteristics of the basin thus the NARX model does not represent the basin system. Moreover, the major criticism towards data-driven models, including NARX, is the physical meaningless and implicit features (Jimeno-Sáez et al. 2018). Data-driven models only make predictions for selected points (Srivastava et al. 2006). In addition, due to the sensitivity of these models to the value of outliers in the training process, it would be more appropriate to use them in the macro perspective (Zakizadeh et al. 2020).

### Comparison of SWAT and NARX models

In this study, the results from the physically based and data-driven models were compared for predicting reservoir volumes and streamflow at the Tersakan Basin. We compared the training period results from the NARX model with the calibration period results from the SWAT model. Additionally, we compared the validation/training results from the NARX model with the validation period results from the SWAT model. In model comparison, we used five different metrics, *R*^2^, RMSE, NRMSE, KGE, and NSE. According to the model performances provided in Tables 5 and 6, the NARX model provided better performance than the SWAT model for reservoir volume and streamflow prediction based on all five metrics.

There is no study in the literature comparing NARX and SWAT models. However, there are some studies for streamflow prediction with other data-driven models, such as ANNs, LSTM, and SWAT models, that were used together (Table 1). The performance of the ANN model was compared with SWAT in many studies (Ahmadi et al. 2019; Demirel et al. 2009; Fan et al. 2020; Jimeno-Sáez et al. 2018; Kim et al. 2015a; Makwana and Tiwari 2017; Pradhan et al. 2020; Rabezanahary Tanteliniaina et al. 2021; Srivastava et al. 2006; Valeh et al. 2021; Wagena et al. 2020; Zakizadeh et al. 2020). Some other studies compared the performance of the SWAT with SOM (Kim et al. 2015a), XGBoost (Ji et al. 2021), SVR (Ji et al. 2021), and LSTM (Giha et al. 2018; Ji et al. 2021; Kim and Kim 2021; Lee et al. 2020). In these studies, *R*^2^ values calculated with SWAT ranged from 0.49 to 0.92, while those with the data-driven models were between 0.52 and 0.98. The NSE values calculated based on simulations with SWAT ranged from 0.47 and 0.90, and they were between 0.49 and 0.98 with the data-driven models. The results of this study were compatible with the previous studies for streamflow prediction. These studies showed that the performances of the data-driven models were better than the SWAT model in estimating streamflow. However, they also emphasized that the SWAT model provides the hydrological water balance better, while the ANN model produces the result without considering the hydrological outputs.

Results showed that with both models, the best performance was obtained when predicting water volumes at the Ladik reservoir. The second best performance was obtained during prediction of water volumes at the Yedikir reservoir. The model performances were the lowest for prediction of streamflow at the basin outlet Ladik Reservoir is located, where the Tersakan River starts in the basin. Yedikir reservoir is located close to the basin outlet (Fig. 3). Agricultural areas are denser towards the basin outlet and there is a lot of uncontrolled irrigation here. These results suggest that both model performances are more affected where human influences are intense (Özdoğan-Sarıkoç et al. 2023).

As SWAT model includes the physical conceptualization of the watershed and simulates processes that affect water movement, it can produce information about water balance, which is useful for understanding hydrological processes. In addition, the SWAT model’s ability to complete the missing data is also among its advantages over the NARX model (Makwana and Tiwari 2017). The weaknesses of the SWAT model are that it requires a lot of data for model development and is time consuming, and variable selection is difficult and requires expertise (Zakizadeh et al. 2020). On the other hand, NARX model is that it is easier to implement, as it does not require any physical properties in the basin. It requires less cost and data and is better and faster than SWAT. It is more suitable to use for basins where necessary data for establishing a physically based model are limited, such as the Tersakan Basin. However, it is one of the important criticisms that it is a black-box and produces physically meaningless results (Jimeno-Sáez et al. 2018). NARX model also does not represent a watershed system in the spatial dimension and therefore cannot make predictions at various points along the stream. It can perform prediction only at the point where it is informed (Srivastava et al. 2006). However, SWAT model can calculate parameters for each sub-basin and predict inflows and outflows for each sub-basin (Zakizadeh et al. 2020). Another shortcoming of NARX model is that they need to be retrained for data changes in the watershed. This indicates that they cannot be used to predict future conditions associated with the watershed.

### Limitations and future work

The SWAT model is created based on the physical characteristics of the watershed area, which requires extensive information about topography, soils, and land use/cover. Unfortunately, the physical data about the Tersakan basin were quite limited; therefore, data from global datasets were to be used. If high-quality and high-resolution data could have been reached, the performance of the SWAT model could have been better (Ahmadi et al. 2019). Also this study used data from a single meteorological station as the stations available in the basin did not provide regular and long-term data. This might have prevented the inclusion of some local climatic events in the simulations (Thodsen et al. 2017). In the future, the availability of data from global datasets that offer higher spatial and temporal resolutions and meteorological data from multiple stations could improve the performance of the SWAT model. Due to presence of reservoirs in the basin, we used a watershed configuration consisting of 8 sub-basins and 220 HRUs. Different watershed configurations could have been created with different number of sub-basins/HRUs. In the future, the effects of number of sub-basins/HRUs on the model performance could be evaluated.

When two models are evaluated according to the same performance evaluation criteria, the NARX model performance was better than that of the SWAT model. However, here we should note that the performance criteria used in this study (i.e., Moriasi et al. (2015)) was developed for physically based models. Data-driven models usually yield higher performance. The suitability of performance criteria for data-driven models should be evaluated in the future.

Both physically based and data-driven models have advantages under different conditions. Combining data-driven models with physically based models can strengthen these advantages and lower the shortcomings and has the potential to produce a superior hydrological output. Some examples to the use of hybrid models is using the outputs from physically based model as inputs to the data-driven model (Noori and Kalin 2016; Wang et al. 2022) or using data-driven models for improving the quality of inputs to the data-driven model (Liang et al. 2017). In the future, we plan to examine alternative configurations using both approaches.

## Conclusions

Physically based models are used to understand various hydrological processes and to predict the behavior of systems. These models are a function of various parameters used to describe watershed features and produce a set of equations that are used to predict reservoir volume and streamflow. On the other side, data-driven models provide good alternatives without prior knowledge about physical processes. In these models, the representation of model output is more important than representing watershed processes. Creating physically based hydrological models requires expertise, and the process is more complex and lengthy but data-driven models are created much easily and have strong learning capabilities.

The main results of this study can be summarized as follows:

1. Both models produced satisfactory results in the estimation of reservoir volume and streamflow. However, according to the performance evaluation criteria, the NARX model produced better results than the SWAT model.
2. The SWAT and NARX models were both provided the best performance when predicting water volumes at the Ladik reservoir. The second best performance with both models was obtained during prediction of water volumes at the Yedikir reservoir. The model performances were the lowest for prediction of streamflow at the basin outlet. We argue that the degree of human intervention on the hydrologic system increased from upstream to downstream in the basin and these interventions affected model performance.
3. The SWAT model is a physically based model that simulates hydrological processes in the basin. The NARX model, on the other hand, is a result-based model and it does not focus on processes. Therefore, the comparison of these models is challenging due to their different nature. In this study, the NARX model provided better results than the SWAT model. However, the SWAT model could have produced more reasonable results for predicting reservoir volume or streamflows in the future and could better adapt to changing physical or hydrological conditions as it represent the basin processes better.

In general, this study shows that there is no single better model for predicting reservoir volume and streamflow. Each approach has its own weaknesses and strengths. Hence, in future studies, we suggest hybrid models, which combines with physically based and data-driven models, can be used.

## Data Availability

The authors confirm that the data supporting the findings of this study are available within the article.
